# Reference values for gait temporal and loading symmetry of lower-limb amputees can help in refocusing rehabilitation targets

**DOI:** 10.1186/s12984-018-0403-x

**Published:** 2018-09-05

**Authors:** Andrea Giovanni Cutti, Gennaro Verni, Gian Luca Migliore, Amedeo Amoresano, Michele Raggi

**Affiliations:** INAIL Prosthetic Center, Via Rabuina 14, 40054 Vigorso di Budrio, BO Italy

**Keywords:** Gait, Ground reaction force, Symmetry, Rehabilitation, Amputees, Prosthesis, Osteoarthritis, C-leg, Microprocessor controlled knees, Energy storage and return feet

## Abstract

**Background:**

The literature suggests that optimal levels of gait symmetry might exist for lower-limb amputees. Not only these optimal values are unknown, but we also don’t know typical symmetry ratios or which measures of symmetry are essential. Focusing on the symmetries of stance, step, first peak and impulse of the ground reaction force, the aim of this work was to answer to three methodological and three clinical questions. The methodological questions wanted to establish a minimum set of symmetry indexes to study and if there are limitations in their calculations. The clinical questions wanted to establish if typical levels of temporal and loading symmetry exist, and change with the level of amputation and prosthetic components.

**Methods:**

Sixty traumatic, K3-K4 amputees were involved in the study: 12 transfemoral mechanical knee users (TFM), 25 C-leg knee users (TFC), and 23 transtibial amputees (TT). Ninety-two percent used the Ossur Variflex foot. Ten healthy subjects were also included. Ground reaction force from both feet were collected with the Novel Pedar-X. Symmetry indexes were calculated and statistically compared with regression analyses and non-parametric analysis of variance among subjects.

**Results:**

Stance symmetry can be reported instead of step, but it cannot substitute impulse and first peak symmetry. The first peak cannot always be detected on all amputees. Statistically significant differences exist for stance symmetry among all groups, for impulse symmetry between TFM and TFC/TT, for first peak symmetry between transfemoral amputees altogether and TT. Regarding impulse symmetry, 25% of TFC and 43% of TT had a higher impulse on the prosthetic side. Regarding first peak symmetry, 59% of TF and 30% of TT loaded more the prosthetic side.

**Conclusions:**

Typical levels of symmetry for stance, impulse and first peak change with the level of amputation and componentry. Indications exist that C-leg and energy-storage-and-return feet can improve symmetry. Results are suggestive of two mechanisms related to sound side knee osteoarthritis: increased impulse for TF and increased first peak for TT. These results can be useful in clinics to set rehabilitation targets, understand the advancements of a patient during gait retraining, compare and chose components and possibly rehabilitation programs.

## Background

Lower-limb amputees tend to walk asymmetrically when looking at gait temporal and loading parameters, with more time spent and load exerted on the intact limb [1–9]. Temporal asymmetry is typically measured based on step or stance duration; loading asymmetry based the magnitude of the first peak of the vertical ground reaction force (GRF), and the impulse of GRF [2, 3, 6, 10].

Temporal and loading asymmetries were associated to several comorbidities [5]: increased falls [11], osteoarthritis of the sound limb [10, 12–15], osteoporosis of the contralateral limb [15, 16], back pain [17–20]. In addition, walking in public with noticeable asymmetries attracts the general attention [21], which can be very uncomfortable for some prosthesis users. With this background, it is not surprising that a common, almost unquestioned [22], goal for rehabilitation is to regain a symmetric walking [9, 23].

However, the literature does not clearly indicate that striving for perfect symmetry is really and always the best option. Already in 1998, Winter & Sienko [1] stated that “human system with major structural asymmetries in the neuromuscular skeletal system cannot be optimal when gait is symmetrical. Rather, a new non-symmetrical optimal is probably being sought by the amputee within the constraints of his residual system and the mechanics of his prosthesis”. Later in 2005, Schmid and co-workers [3] compared the center of pressure trajectories under the sound and prosthetic foot of transfemoral amputees and concluded that the longer stance on the sound side can be ascribed to the greater ability of the sound leg to advance the step and maintain balance until the prosthetic limb can sustain the body weight. Hof et al. [4] corroborated this explanation in the theoretical framework of the “extrapolated center of mass” [24], concluding that stance time asymmetry is a “sensible adaptation” of experienced transfemoral amputees to improve stability during walking, to overcome the missing lateral ankle strategy of prosthetic feet. More recently, Adamczyk & Kuo [8], with a theoretical and experimental approach involving transtibial amputees, concluded that “some asymmetry may be unavoidable in cases of unilateral limb loss” due to the reduced ankle plantar flexion of the ankle, with direct consequences on stance duration, greater collision work at the sound side, greater work overall, and increased peak force at loading response [25–27]. Imposing symmetry can actually be detrimental, as also observed by [27, 28].

The evidences from the literature, therefore, indicate that optimal symmetry ratios might exist, to obtain a compromise among stability, forward progression, preservation of body structures and perception of a “normal and symmetric biped locomotion” [21]. Unfortunately, at present not only optimal symmetry ratios are unknown, but we also don’t know typical symmetry ratios or which measures of symmetry are essential and which are redundant.

In our opinion, 3 methodological and 3 clinical questions should be answered to clarify these open issues. The *methodological* questions are:

- Q1: do all amputees show the typical M-shaped pattern of the GRF [29], with presence and appropriate timing of its two peaks? In case of a negative answer, the measure of loading symmetry based on the first peak of GRF will be restrict to patients presenting the M-shaped pattern;
- Q2: can we limit the study of temporal symmetry to stance, leaving out step symmetry? We will give a positive answer if stance and step symmetries are very strongly correlated for all amputees, with a coefficient of determination *R*^*2*^ > 0.64 [30];
- Q3: can we further limit the study of gait symmetry to just stance symmetry, leaving out loading symmetry, whose measure requires more cumbersome and expensive equipment? We will give a positive answer if stance symmetry is very strongly correlated (*R*^*2*^ > 0.64) with the symmetry of the first peak and impulse of GRF.

The *clinical* questions are:

- Q4: does gait symmetry depend on the level of amputation? In case of a positive answer, typical ranges of symmetry should be established, which can be used to understand how far a new patient is from well adapted prosthesis users in terms of percentiles;
- Q5: do advanced prosthetic components improve temporal and loading symmetry? In particular, do C-leg users have better results than mechanical knee users of the same mobility level?
- Q6: is it always true that amputees overload the sound side both in terms of first peak and impulse of GRF, thus contributing to the development of osteoarthritis?

Unfortunately, at present it is difficult to answer to these questions based on the available literature, because there are no studies that considered, *at the same time* 1) both temporal and loading asymmetries, 2) both transfemoral and transtibial amputees treated at the *same* prosthetic & rehabilitation center, 3) mechanical and electronic knees, 4) energy-storage-and-return feet instead of the SACH (Solid-Ankle Cushion-Heel) foot. Moreover, the number of patients included is typically limited to 8, both for studies on transtibial and transfemoral amputees. Finally, no studies addressed the correlation between temporal and loading parameters.

The aim of this study was to overcome these limitations and answer to questions Q1-Q6 on three groups of well-adapted, traumatic, K3-K4 amputees: transfemoral amputees using a restricted set of mechanical knees (TFM), transfemoral amputees using the C-leg (TFC), transtibial amputees using energy-storage-and-return feet (TT). A additional group of healthy control subjects (“Controls” in short), was also included to highlight general trends.

## Methods

### Subjects

Sixty K3-K4 lower-limb amputees participated in the study after signing an informed consent: 12 mechanical knee users (TFM, 46 ± 10 y.o.), 25 C-leg users (TFC, 48 ± 13 y.o), 23 transtibial amputees (TT, 44 ± 14 y.o.), with no statistically significant differences in term of age (ANOVA, *p* > 0.62). Ten controls were also included (28 ± 2 y.o.). All amputees had completed a 3-week, intense gait training program at the same specialized prosthetic & rehabilitation center, with the support of the same rehabilitation team. The clinical center has ISO 9001 treatment pathways for amputees and provides over 800 transfemoral and 1200 transtibial prostheses every year. Following training, all patients had been successfully using their prostheses for at least 1 month at the time of testing.

The components provided to patients are summarized in Table 1. Almost 92% of patients used either the Variflex or Variflex LP foot. Mechanical knees were selected to match the activity level of the C-leg, and are consistent with knees selected for comparison with the C-leg in previous studies [31, 32].

**Table 1:** Prosthetic components used and associated quantities

	TFM	TFC	TT
Foot	Variflex LP: 10 1C40: 2	Variflex LP: 25	Variflex: 18 Variflex LP: 2 Truestep: 1 Esprit: 1 1C40: 1
Knee	TotalKnee 2100: 5 3R60: 2 Mauch: 2	C-leg: 25	

### Measurements

After standing still for 10 s, subjects walked along a long indoor hall at self-selected speed, that was noted. During this trial, the GRF was measured on each side through instrumented insoles (Pedar-X, Novel, D), sampling at 100 Hz [33, 34].

### Data processing

For each subject, GRF data were export to MATLAB. Based on the 10 s’ orthostatic posture, body weight was calculated. Assuming a foot-floor contact threshold at 10% body weight, we detected heel-strike and toe-off events for the two sides. We isolated the steady state condition by considering the central 10 strides.

#### Calculation of temporal symmetry

For each stride, we calculated the step and stance duration. Then, for each couple of consecutive sound-affected gait cycles, we calculated the following indexes of symmetry:

- Step Symmetry (SPS): Step Duration _*SOUND*_ / Step Duration _*AFFECTED*_
- Stance Symmetry (SNS): Stance Duration _*SOUND*_ / Stance Duration _*AFFECTED*_

For Controls, ratios were right over left side. A value of 1 represents perfect symmetry. For each index of symmetry, we calculated the subject’s median value over the trial. Finally, we obtained the distribution of the median values for the two indexes over TFM, TFC, TT and Controls.

#### Calculation of loading symmetry

For each gait cycle, the integral over the stance period of GRF was calculated, i.e. the *impulse of GRF*, as previously reported by [2]. Then, for each couple of consecutive sound-affected gait cycles, we calculated the index of symmetry:

- Impulse Symmetry (IMS): Impulse _*SOUND*_ / Impulse _*AFFECTED*_

A value of 1 represents perfect symmetry. Right over left side was used for Controls.

Afterward, the GRF profile of each gait cycle was checked to verify the presence of the first peak within the 0–40% of the gait cycle, and of a second peak within the 60–100%. Subjects reporting both peaks in more than half of the trials formed the “*Two-Peaks”* subgroup.

For the subjects in *Two-Peaks* we operated as follows. For each couple of consecutive sound-affected gait cycles, we calculated the following index:

- First Peak Symmetry (P1S): First peak _*SOUND*_ / First peak _*AFFECTED*_

P1S provides a measure of peak force asymmetry at loading response, while IMS provides a measure of the asymmetry in cyclic loading. These are two different mechanism of osteoarthritis development [10, 35–37].

For each index of symmetry, we calculated the subject’s median value over the trial. Finally, we obtained the distribution of the median values for the two indexes over TFM, TFC, TT and Controls.

### Statistical analysis

The distribution of the four indexes of symmetry (SPS, SNS, IMS and P1S) was checked for normality within each group (TFM, TFC, TT and Controls) and over all subjects, both visually with the Normal Probability Plot and with the Lilliefors test. This last failed for SPS_TFM_ and P1S_TT_ and there were doubts about IMS in general.

The relationship between SNS and the three indexes SPS, IMS and P1S was evaluated with regression methods with the MATLAB Curve Fitting Toolbox. The strength of the relationship was primarily evaluated in terms of *R*^*2*^. This statistical parameter, multiplied by 100, is usually interpreted as the variance of “y” accounted for by “x”, where in this case “y” is SPS or IMS or P1S, and “x” is SNS. In addition, the root-mean-square error (RMSE) of the residuals was also reported.

Distributions were reported in terms of median and interquartile range [3], with box plots. For each symmetry index, the Kruskal-Wallis test (α = 0.05) was adopted to check for overall statistically significant differences among TFM, TFC, TT and Controls. In identifying pairwise differences, the Tukey-Kramer “HSD” correction was applied within the MATLAB “multcompare” function.

## Results

Gait speed was compared among TFM (1.12 ± 0.13 m/s), TFC (1.17 ± 0.12 m/s), TT (1.23 ± 0.19 m/s) and Controls (1.41 ± 0.21 m/s). ANOVA did not show statistically significant differences among amputees (*p* = 0.14), but only between Controls and amputees (*p* = 0.0005).

Further results are reported hereinafter based on their relevance for questions Q1-Q6.

### Question Q1

**Fig. 1:**
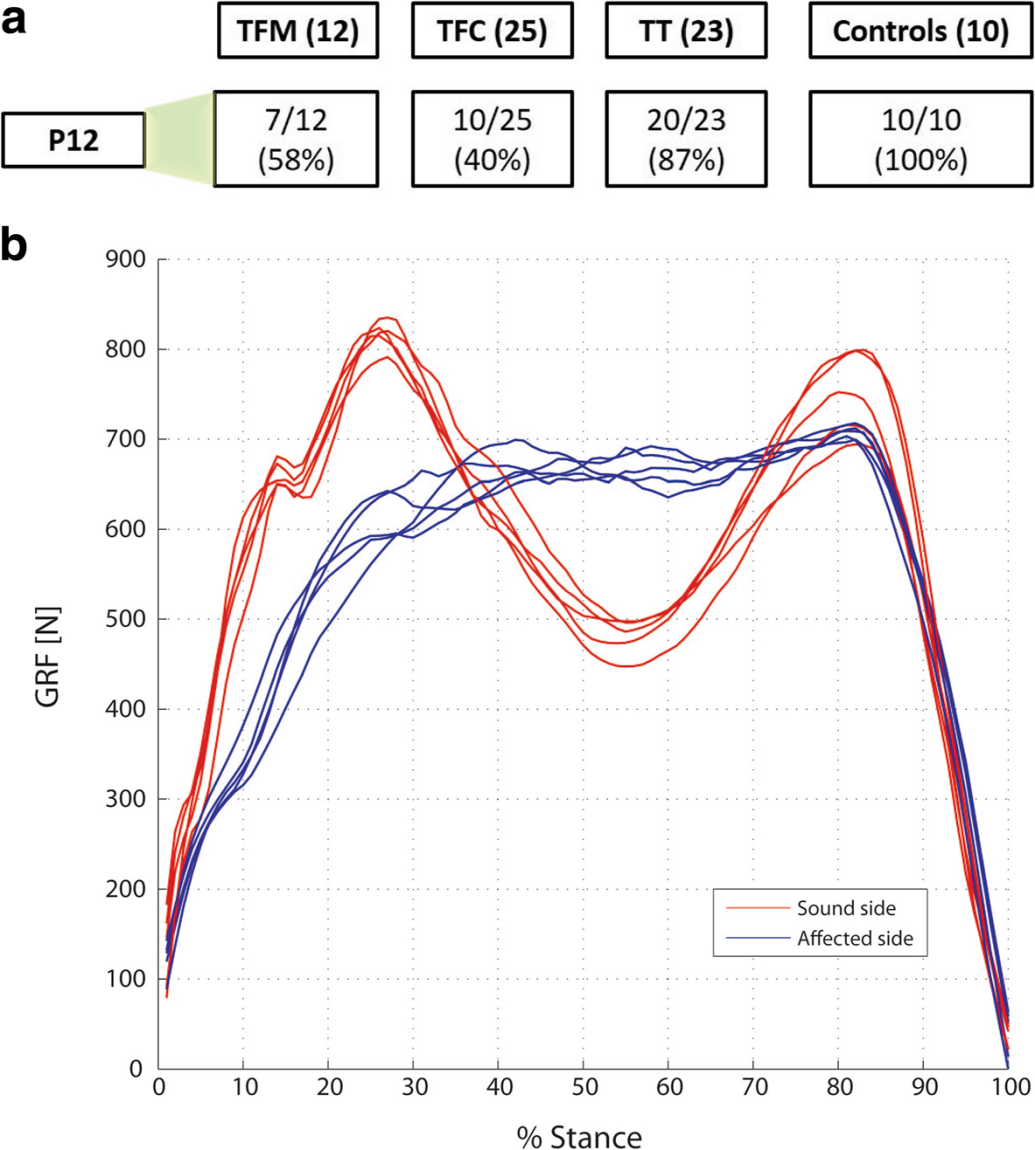
**a** Number of subjects in subgroup *Two-Peaks* for TFM (transfemoral mechanical knee users), TFC (transfemoral C-leg users), TT (transtibial amputees), and Controls: **b** typical alternative vertical ground reaction force pattern shown by TFC patients not included in *Two-Peaks*

Figure 1 reports the number of subjects in subgroups *Two-Peaks*, which decreases from TT (20/23), to TFM (7/12) to TFC (10/25). The number of TFC with non-standard GRF is remarkably high (60%); these patients report a consistent “alternative” pattern (example provided in Fig. 1b). Based on these results, the answer to Q1 was negative and the calculation of the symmetry index P1S was restricted to the subjects in *Two-Peaks*.

### Question Q2

**Fig. 2:**
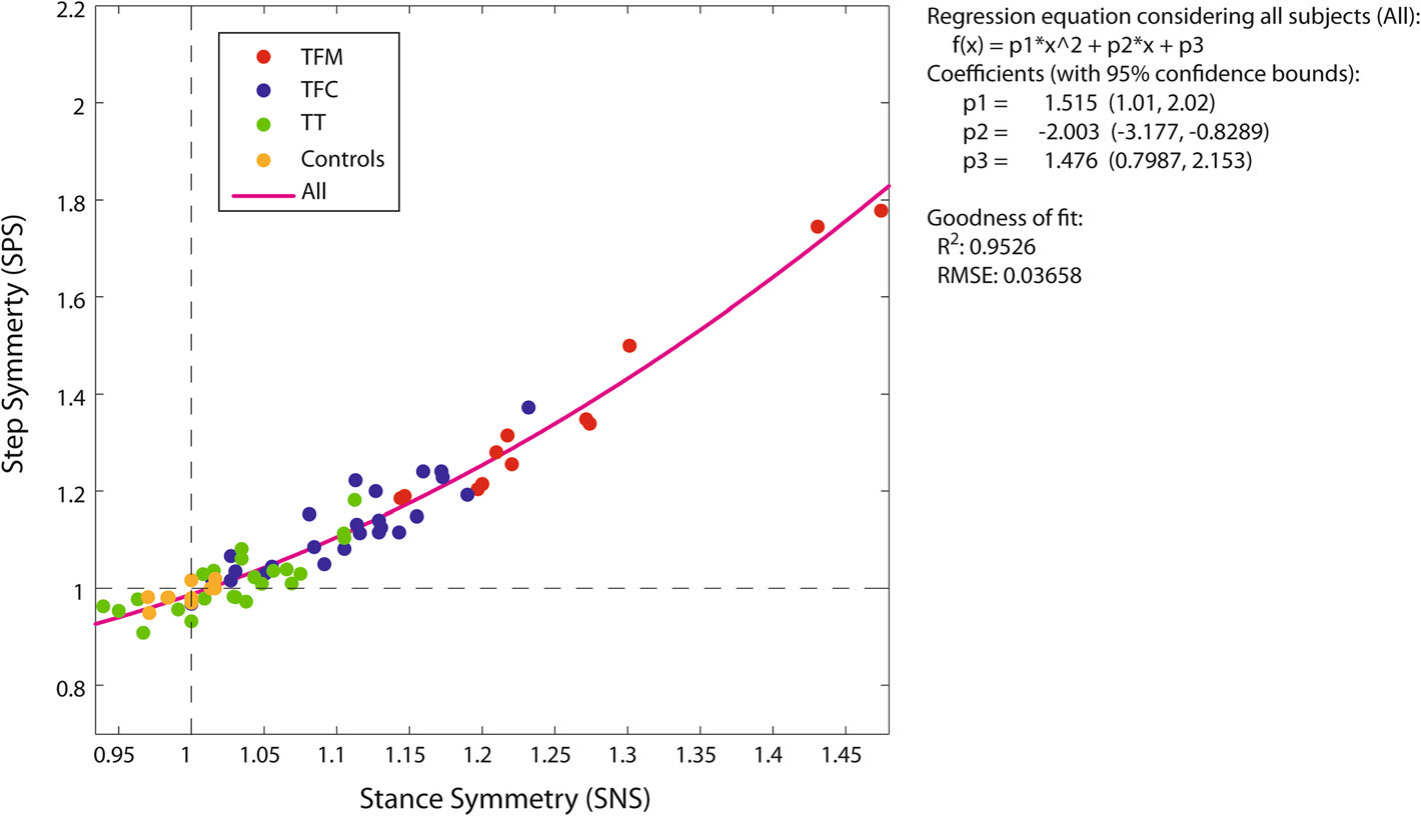
Step symmetry index (SPS) vs Stance symmetry index (SNS). Each dot represents one subject. Subjects of the same group feature the same color (see legend in the plot). The purple parabolic line is the regression line for ALL subjects together. The equation of the fitting is reported on the right, with the fitting quality parameters *R*^*2*^ (coefficient of determination) and RMSE (Root Mean Squared Error)

Figure 2 reports the regression analysis for SPS vs SNS considering the whole set of patients and Controls (“ALL” in brief). *R*^*2*^ and RMSE values for each group (TFM, TFC, TT, Controls) and ALL are reported in Table 2. *R*^*2*^ was at least 0.70 for all amputees, with RMSE < 0.042. Therefore, the answer to Q2 was positive and only SPS was further considered.

**Table 2:** Quality of fit of the regressions for step (SPS), impulse (IMS) and first peak symmetry (P1S) indexes vs stance symmetry index (SNS)

	SPS vs SNS		IMS vs SNS		P1S vs SNS	
	*R* ^*2*^	*RMSE*	*R* ^*2*^	*RMSE*	*R* ^*2*^	*RMSE*
TFM	**0,97**	**0,042**	**0,81**	**0,137**	*0,06*	*0,154*
TFC	**0,81**	**0,042**	**0,69**	**0,090**	*0,04*	*0,177*
TT	**0,70**	**0,036**	0,37	0,128	*0,20*	*0,289*
CONTROLS	0,51	0,017	0,47	0,040	*0,05*	*0,038*
ALL	**0,95**	**0,037**	**0,79**	**0,103**	*0,00*	*0,247*

The coefficient of determination (*R*^*2*^), and the Root Mean Squared Error (RMSE) are reported for every group (*TFM* transfemoral mechanical knee users, *TFC* transfemoral C-leg users, *TT* transtibial amputees, Controls), and for all subjects altogether (ALL). Bold: *R*^*2*^ > 0.64, Regular: 0.36 < *R*^*2*^ *< 0.64*, Italic: *R*^*2*^ < 0.36 [30]

### Question Q3

**Fig. 3:**
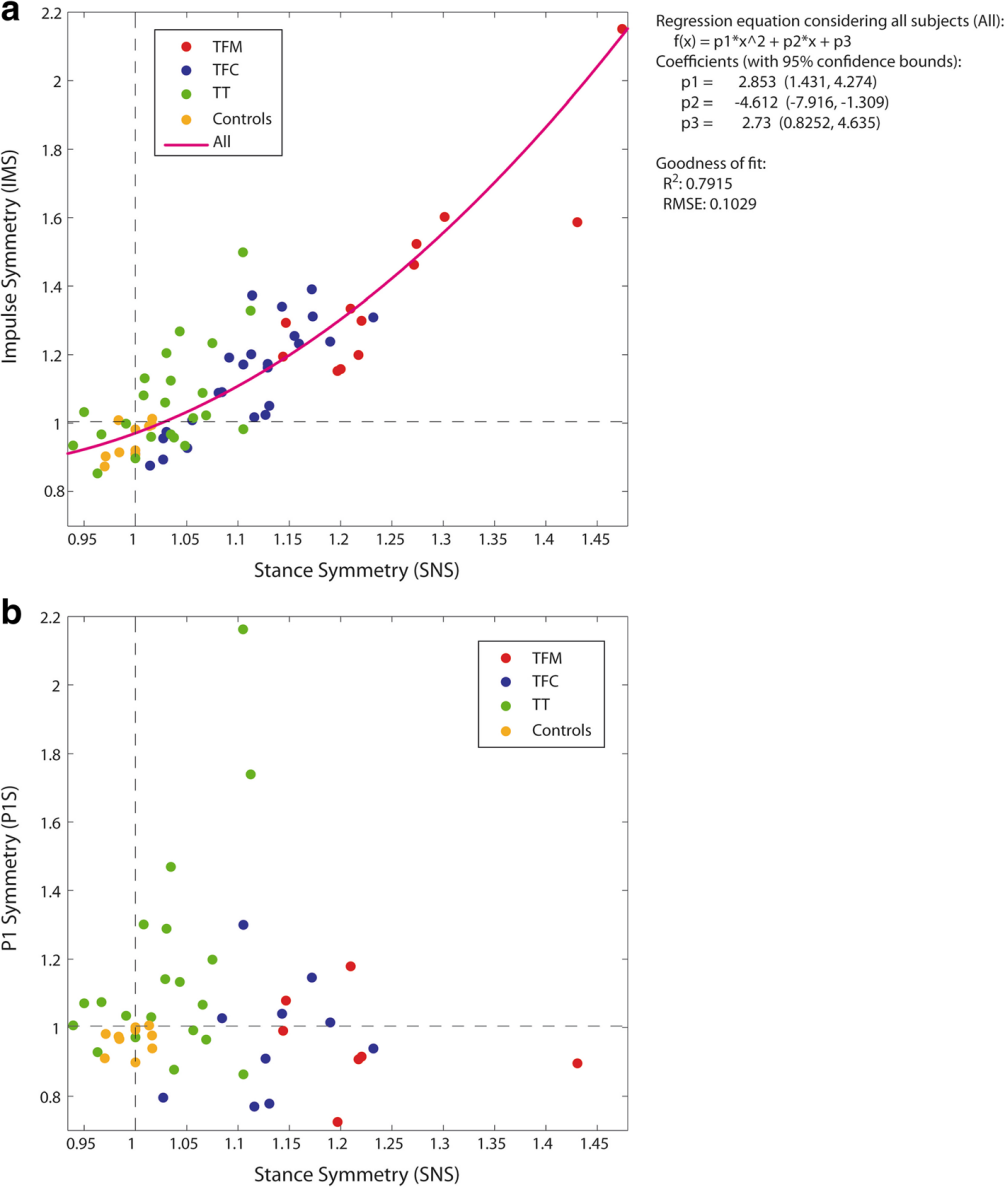
**a** Impulse symmetry index (IMS) vs Stance symmetry index (SNS) and **b** First peak symmetry index (P1S) vs SNS. Each dot represents a subject. Subjects of the same group feature the same color (see legend in the plot). In (**a**), the purple parabolic line is the regression line for ALL subjects together. The equation of the fitting is reported on the right, with the fitting quality parameters *R*^*2*^ (coefficient of determination) and RMSE (Root Mean Squared Error). No valid regression was found for P1S vs SNS. TFM: transfemoral mechanical knee users, TFC: transfemoral C-leg users, TT: transtibial amputees

Figure 3a and b report the regression analysis for IMS vs SNS and P1S vs SNS, respectively, for ALL. *R*^*2*^ and RMSE values for each group (TFM, TFC, TT, Controls) and ALL are reported in Table 2. For IMS vs SNS, *R*^*2*^ was lower than 0.64 for TT, with RMSE > 0.128. For P1S vs SNS, *R*^*2*^ was lower than 0.2 for all amputees. Therefore, the answer to Q3 was negative and IMS, P1S and SPS were separately considered in all subsequent analyses.

### Questions Q4-Q6

**Fig. 4:**
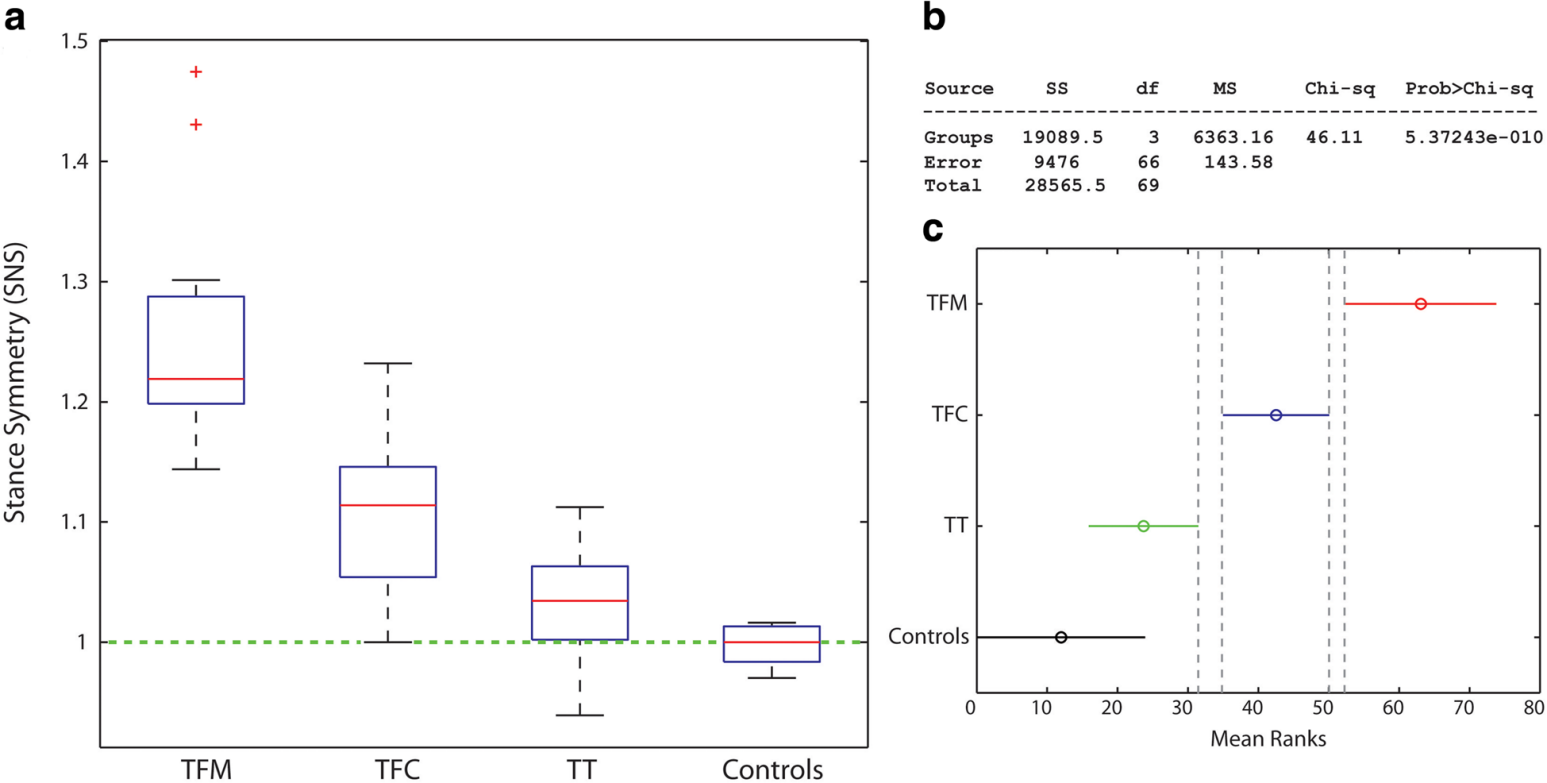
**a** Box plot for the stance symmetry index (SNS) over the groups; **b** Results of the Kruskal-Wallis test; **c** Pairwise comparisons: non-overlapping lines indicate a statistically significant difference. TFM: transfemoral mechanical knee users, TFC: transfemoral C-leg users, TT: transtibial amputees

**Fig. 5:**
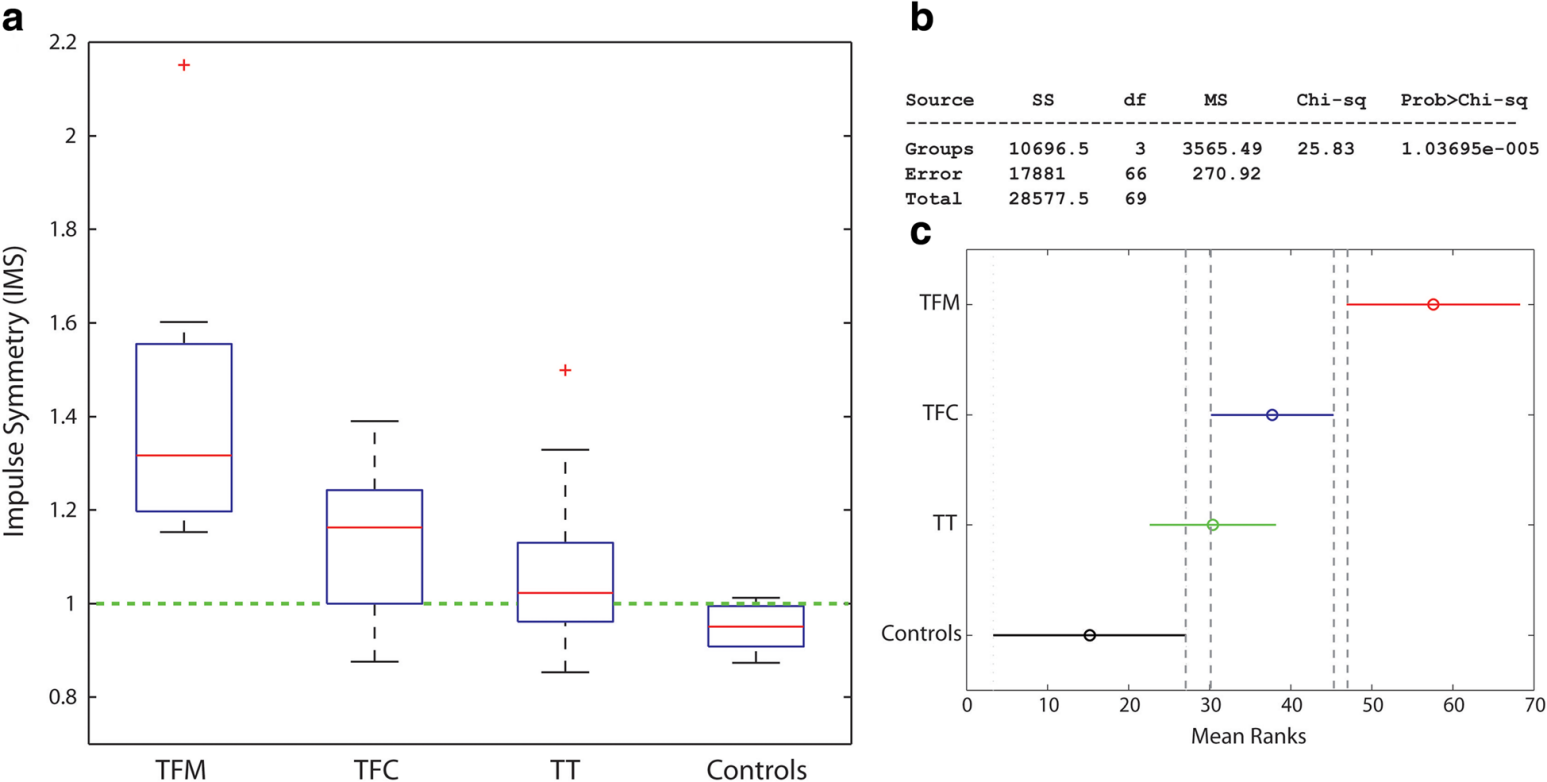
**a** Box plot for the impulse symmetry index (IMS) over the groups; **b** Results of the Kruskal-Wallis test; **c** Pairwise comparisons: non-overlapping lines indicate a statistically significant difference. TFM: transfemoral mechanical knee users, TFC: transfemoral C-leg users, TT: transtibial amputees

**Fig. 6:**
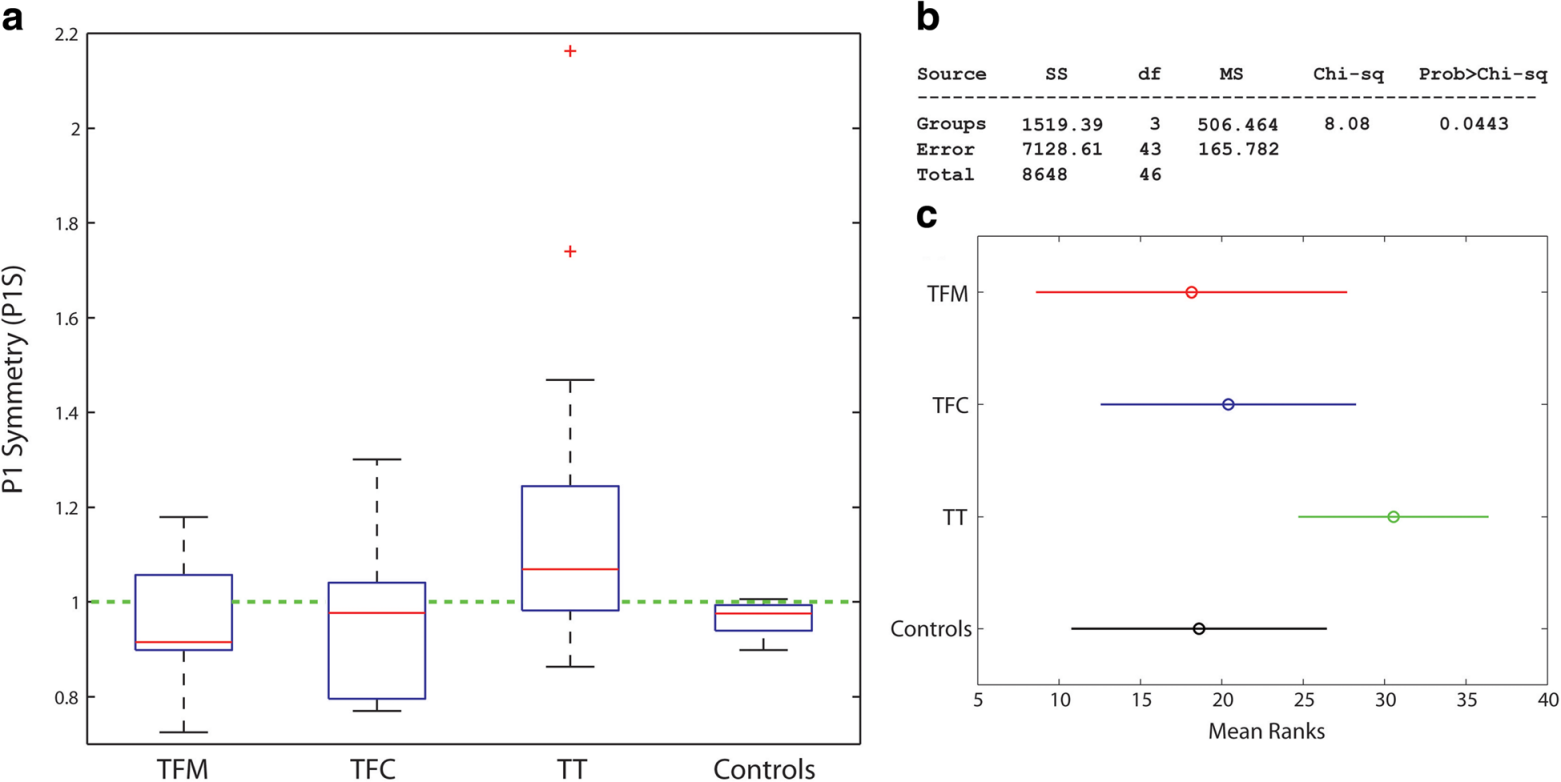
**a** Box plot for the first peak symmetry index (P1S) over the groups; **b** Results of the Kruskal-Wallis test; **c** Pairwise comparisons: non-overlapping lines indicate a statistically significant difference. TFM: transfemoral mechanical knee users, TFC: transfemoral C-leg users, TT: transtibial amputees

Figures 4a, 5a and 6a report the distribution of SNS, IMS and P1S for TFC, TFC, TT and Controls. Numerical values are reported in Table 3.

**Table 3:** Numerical values for the indexes of symmetry SNS (stance), IMS (impulse) and P1S (first peak)

	SNS				IMS				P1S			
	Median	25th	75th	IQR	Median	25th	75th	IQR	Median	25th	75th	IQR
TFM	1,22	1,20	1,29	0,09	1,32	1,20	1,55	0,36	0,91	0,90	1,06	0,16
TFC	1,11	1,05	1,15	0,09	1,16	1,00	1,24	0,24	0,98	0,80	1,04	0,24
TF									*0,94*	*0,87*	*1,05*	*0,18*
TT	1,03	1,00	1,06	0,06	1,02	0,96	1,13	0,17	1,07	0,98	1,24	0,26
CONTROLS	1,02	0,98	1,01	0,03	0,95	0,91	0,99	0,09	0,98	0,94	0,99	0,05

For each group, the median is reported together with the 25th, 75th and interquartile range (IQR). *TFM* transfemoral mechanical knee users, *TFC* transfemoral C-leg users, *TF* transfemoral, *TT* transtibial amputees

For SNS and IMS, the Kruskal-Wallis test showed statistically significant differences among the medians of the groups (*p* < 0.0001) (Figs. 4b and 5b). The pairwise analyses for:

- SNS (Fig. 4c) showed that all amputee groups are different among each other, supporting a positive answer for *Q4 and Q5*;
- IMS (Fig. 5c) showed a statistically significant difference between TFM and all other groups, with all TFM values > 1 as opposed to TFC and TT. This supports a partially positive answer to *Q4*, a positive answer to *Q5* and a negative answer to *Q6.*

For P1S, the Kruskal-Wallis test reported a statistically significant difference in the medians among groups (*p* = 0.0443) (Fig. 6b). The pairwise comparison did not show differences (Fig. 6c). This is a very possible situation for three reasons:

- the Kruskal-Wallis and pairwise comparisons try to negate different hypotheses;
- we applied a quite conservative multiple comparison strategy (HSD);
- the statistical power is reduced by the decreased number of transfemoral amputees (TF) within *Two-Peaks*.

**Fig. 7:**
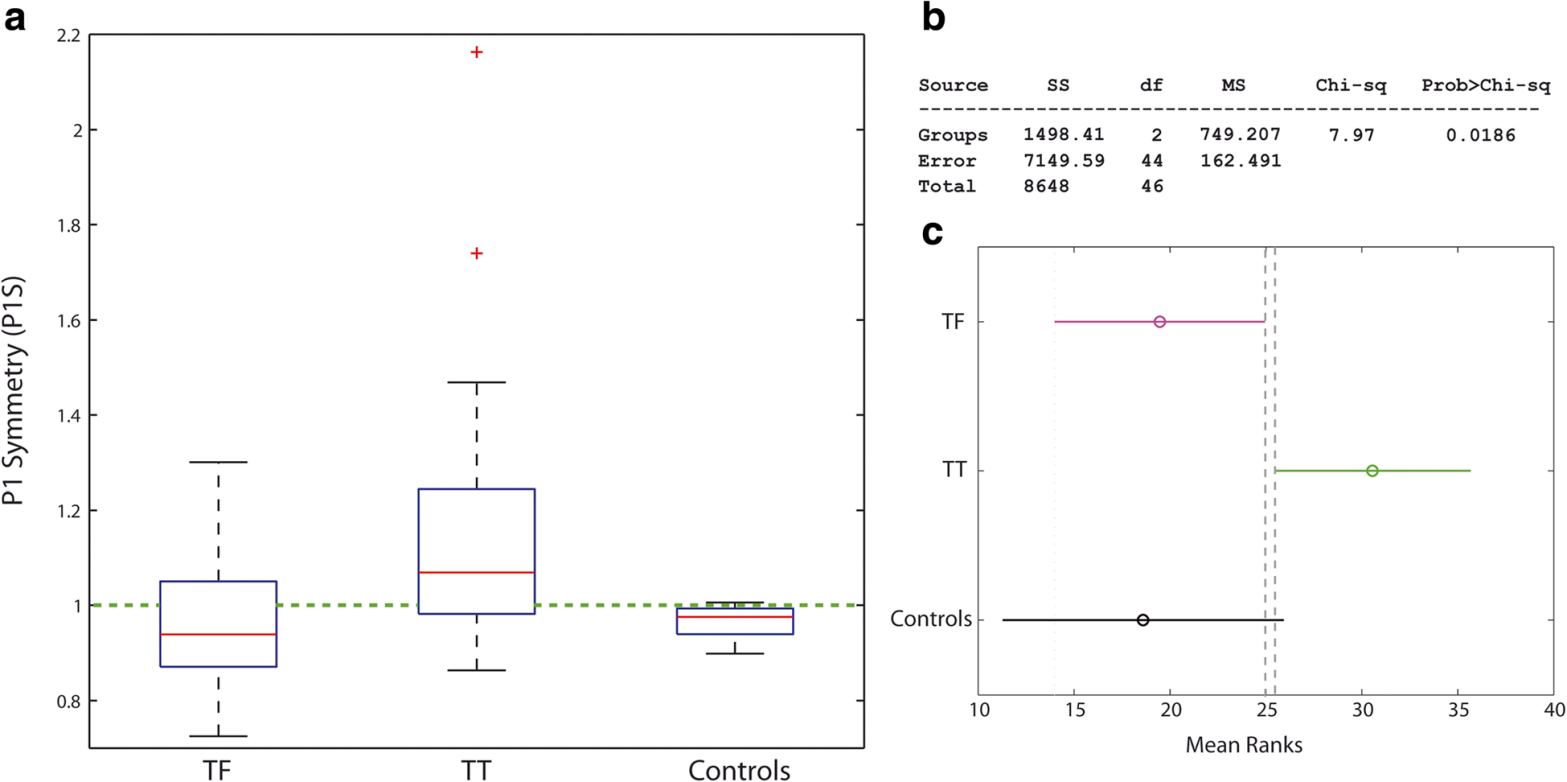
**a** Box plot for the first peak symmetry index (P1S) after grouping all transfemoral amputee together (TF); **b** Results of the Kruskal-Wallis test; **c** Pairwise comparisons: non-overlapping lines indicate a statistically significant difference. TF: transfemoral amputees, TT: transtibial amputees

For this reason, we grouped subjects per level of amputation (TFM and TFC together), and results are reported in Fig. 7a. The Kruskal-Wallis test now shows a stronger significance among groups (*p* = 0.0186) and the pairwise analysis shows a statistically significant difference between TF and TT. The variability in P1S is much higher in amputees than in Controls (Bartlett’s test for equal variances, *p* = 0.001). These results support a negative answer to Q6.

## Discussion

In this study, we addressed three methodological and three clinical questions regarding the temporal and loading symmetry of transfemoral amputees (both mechanical and C-leg users) and transtibial amputees, to support in the development of more targeted rehabilitation goals, that are particularly needed [9, 38].

As a general consideration, the self-selected walking speed was not statistically different among amputees, despite a slight increase in the median from TFM, to TFC, to TT toward Controls. Absolute values compare well with previously reported data [2, 7, 39].

For the sake of clarity, results are discussed below for each question, in comparison with the available literature whenever possible.

### Question Q1

Question Q1 asked if all amputees show the typical M-shaped pattern of the GRF, with presence and appropriate timing of its two peaks. Results support a negative answer.

As previously noted, this is particularly evident for TFC, who presented a consistent “alternative” pattern: after a steep rise (initial contact/loading response), GRF shows a further (almost) monotonical increase (midstance), after which it drops (terminal stance/pre-swing). TFM falling out of *Two-Peaks* did not present this pattern, and were typically not included in *Two-Peaks* due to a delayed P1 after 40% of the stance phase. Since no kinematic and kinetic data were collected, we can just speculate that this TFC pattern is the combined effect of:

- the Variflex behavior, with strong energy storage in loading response [25, 26];
- C-leg knee flexion in loading response [40];
- the confidence gained by this group of amputees on the capacity of the C-leg to sustain them at heel-strike and loading response, with no need to force extension.

The ultimate effect for this pattern is a “soft landing” on the prosthetic side, which might increase comfort [32]. These speculations require future experimental confirmations, but match well with previous evidences that only a fraction of transfemoral amputees can fully rely on C-leg stability despite knee flexion during early stance [32, 40]. This might be the effect of a specialized rehabilitation.

### Question Q2

Question Q2 asked if we can limit the study of temporal symmetry to stance leaving out step symmetry. Results support a positive answer.

The regression of SPS vs SNS for each group and for ALL was quadratic, with excellent fits.

SNS explained from 70 to 97% of the variance in SPS data in amputees (*R*^*2*^, as reported in Table 2). Even for Controls, who feature a very small peak-to-peak SNS (.97 to 1.01), the explained SPS variance is 50% with a RMSE as small as 0.017.

This is the first time that the SPS vs SNS regression is reported in the literature and that a quadratic relationship is described. The nonlinear fit is not surprising, because SPS is non-linearly related to the interplay of 1) the sounds and affected side stance durations and 2) the two double support durations. The quadratic fit stresses the importance of stance time symmetry, since it influences step asymmetry by a factor 2.

### Question Q3

Question Q3 asked if the study of gait symmetry can be limited to just stance temporal symmetry, leaving out loading symmetry. Results support a negative answer.

When IMS vs SNS was examined considering the full set of subjects, a quadratic fit emerged: SNS explained as much as 79% of the variance in IMS. This is the first time this relationship is examined and reported. Since IMS is the integral of GRF over the stance phase, it is not surprising that IMS and SNS are related: a high stance time asymmetry is a leading factor for a high impulse asymmetry. However, GRF magnitude does not linearly increase with time, and has a shape which can differ between the sound and affected side. When all these elements become part of a ratio, it is not surprising that the relation between IMS and SNS can be non-linear.

This conclusion is valid for TFM and TFC at group level too, given the *R*^*2*^ > 0.64. However, this is just partially true for TT, because *R*^*2*^ decreases to 0.37 and the RMSE is high (0.128): reporting SNS and not IMS can be misleading. This different evidence for TT can be ascribed to two factors only:

- The improvement in SNS asymmetry (1.03, IQR 0.06) compared to TFM (1.22, IQR 0.09) and TFC (1.11, IQR 0.09) (Table 3);
- A greater *asymmetry* in GRF magnitude between sides. This is supported by the evidences for P1S, as reported in Fig. 7. Further discussions are postponed to *Q6* below.

An adequate regression for P1S vs SNS was not found for none of the groups and ALL: the two indexes must measure different construct and therefore they must be separately reported.

### Question Q4

Question Q4 asked if gait symmetry depends on the level of amputation. Results support a positive answer.

With reference to SNS, all amputee groups had statistically different median values. All TF spend more time on the sound side: TFM have the highest asymmetry (median asymmetry of 22%), which is twofold the TFC’s (11%). As can be seen in Fig. 4, this is also true for 75% of TT, which means that ¼ of TT do spend more time on the *affected side*. This was never clearly reported in the literature. The TT asymmetry (3%) is 4 times less than TFC. Controls, in median, have a perfect symmetry, with a IQR of just 3%.

The SNS median for TFC (1.11) compares well with the median SNS that can be calculated from the results reported in [3] (1.09). Furthermore, our results can be compared with the study of Nolan et al. [2], that involved 4 transfemoral and 4 transtibial amputees using a single hinge knee and a SACH foot. Once appropriately converted to our indexes, Nolan’s results are reported in Table 4. Results, can also be compared with Bateni et al. [41], which reported a mean stance asymmetry for TT of about 7% (calculated as the ratio of the mean between sides). Compared to these studies, our SNS values are lower. In particular, 63% of TT and 20% of TFC have a SNS lower than ±5%, which makes them unperceived by others as “impaired” walkers with regard to temporal symmetry [21]. This is not surprising given the different prosthetic components used and the fact that our patients followed a specialized rehabilitation training. Our SNS results for TT are also in very good agreement with results reported by Jarvis et al. [38] for young veterans (median 1.04, IQR = 0.03). For TFC, our SNS is higher (1.11 compared to 0.98) but the IQR is much smaller (0.09 compared to 0.20). This remarks that the training for transfemoral amputees is more challenging.

**Table 4:** Results from Nolan et al. [2], converted to the indexes of symmetry used in this study. SNS (stance), IMS (impulse) and P1S (first peak)

	SNS	IMS	P1S
TFM	1,27	1,69	1,22
TT	1,05	1,36	1,25
CONTROLS	1,03	1,08	1,08

Having named *N* the indexes in [2], the new values follow from this equation: *New* = (2 + *N*)/(2-*N*)

*TFM* transfemoral mechanical knee users, *TT* transtibial amputees

When looking at IMS, the TFM median was statistically different from TFC and TT: TFM asymmetry is *twice* that of TFC and *16 times* TT’s. The comparison with Nolan et al. [2] is striking: our TFM had an impulse asymmetry which is half Nolan’s; for TT it is 10 times less. This result points, again, in the direction of the benefits of energy-storage-and-return feet and more advanced knees. Improvement in loading asymmetry with energy-storage-and-return feet and feet with improved roll-over shape has been previously reported in [25, 27, 42], and match well with simulation studies [8].

Finally, P1S results show statistically significant differences between TF and TT (Fig. 7). About 59% of TF have a higher peak on the *prosthetic side*. Our results agree with Castro et al., which did not report an increased peak GRF on the sound side, but rather an increase in the GRF impulse. TT clearly show an asymmetric loading with higher values for the sound side (70% of patients), but 3 times less than that reported by Nolan and co-workers. As previously reported, it is reasonable to ascribe this improvement to the use of energy-storage-and-return feet compared to SACH [27, 43].

### Question Q5

Question Q5 asked if advanced prosthetic components improve temporal and loading symmetry, and if C-leg users have better results than mechanical knee users of the same mobility level. Results support a positive answer.

Results have been partially discussed while addressing Q1 and Q4 and can be summarized stating that TFC were statistically different from TFM for SNS and IMS. Results for IMS bring TFC to undistinguishable results to TT.

Also, the C-leg in combination with Variflex triggers a new GRF pattern that possibly ensures an increased comfort during walking (Question Q1). This requires further experimental confirmations.

Petersen et al. [44] have previously reported about SNS in C-leg users compared to TFM. However, that study was not able to prove a statistically significant improvement but just a trend, probably due to the small number of subjects included (5) with different amputation etiologies. Our results confirm that trend, with statistically significant differences. More generally, a considerable body of knowledge is available about the positive effects of the C-leg on amputees’ mobility [31, 45–47], gait kinematic [32–40], kinetic [39] and step-length symmetry [32]. Our findings match well with this general trend toward improved symmetries.

As discussed in Q4, the comparison of the literature with our results for TT suggests a possible positive effect of energy-storage-and-return feet in comparison with SACH, for all the indexes of symmetry.

### Question Q6

Question Q6 asked if it is always true that amputees overload the sound side both in terms of first peak and impulse of GRF, thus contributing to the development of osteoarthritis. Results support a negative answer.

As previously discussed about Q4, if we focus on IMS, 100% of TFM overload the *sound side*. This percentage decreases to 75% of TFC and 57% of TT. If we look at P1S, 41% TF load more the *sound side*. However, this percentage rises to 70% for TT. Based on these different percentages of TT and TF for IMS and P1S, it could be argued that two different mechanisms might be related to knee osteoarthritis for the two groups: peak overload for TT (measured by P1S), and extended duration of force action (impulse) for TF (measured by IMS). Given the higher prevalence of knee osteoarthritis in TF compared to TT [5, 10], it might be speculated that the second mechanism is more detrimental than the first.

## Conclusions

In the Introduction, we posed three methodological and three clinical questions regarding the gait temporal and loading symmetry of lower-limb amputees. Based on the results collected on traumatic, K3-K4, transfemoral (mechanical knees and C-leg users) and transtibial patients successfully fit and trained in using their prosthesis, we can answer as follows.

The three *methodological* questions wanted to establish a minimum set of symmetry indexes to study and if there are limitations in their calculations. *First*, the first peak of the vertical ground reaction force at loading response cannot be clearly identified in all amputees, and the calculation of its index of symmetry was limited to patients with the typical M-Shaped pattern of the ground reaction force. *Second*, the analysis of temporal symmetry can be limited to stance, leaving out step symmetry. *Third*, stance, impulse and first peak symmetries should be separately reported.

The three *clinical* questions wanted to establish if “typical” levels of temporal and loading symmetry exist and change with the level of amputation and prosthetic components. *First,* the symmetries of stance, impulse and first peak are all influenced by the level of amputation. In particular, the time spent on the sound side decreases significantly from transfemoral mechanical knee users, to C-leg users, to transtibial patients. The impulse on the sound side decreases significantly from mechanical knee users to C-leg and transtibial patients. Transtibial patients have a higher first peak at loading response on their sound side, while most transfemoral patients do not. *Second*, advanced prosthetic component seem to positively influence the temporal and loading symmetry. In particular, the C-leg in combination with the Variflex foot improves stance, impulse symmetry and for about 60% of patients smooths the first peak at loading response. About 20% of C-leg users have a stance asymmetry which is below the level of perceived impaired gait, compared to 0% of mechanical knee users. For transtibial patients, comparisons of our results with the literature point toward an improvement of all indexes of symmetry, possibly due to the use of energy-storage-and-return feet instead of SACH feet. *Third,* it is not always true that amputees overload the sound side. Percentagewise, transfemoral amputees tend to overload the sound side with increased impulse, while TT with increased peak GRF. This might be suggestive of two separate mechanisms for the onset of knee osteoarthritis.

We think that our results can be exploited in the clinical routine. *First*, clinicians can use our results to set reasonable targets for rehabilitation. Specifically, they can compare the level of symmetry of a new patient with the ranges provided, and put the patient’s performance and advancements during rehabilitation in perspective. Moreover, technical and healthcare professionals might use our findings to compare the effect of different prosthetic components and potentially the effect of different rehabilitation programs. *Second*, it is often required by payers (e.g. insurances, public healthcare services, or patients), to justify the use of advanced prosthetic components. We think that our results support the use of C-leg and energy-storage-and-return feet on K3-K4 traumatic patients: thanks to the improvement in temporal and loading symmetry compared to mechanical knees and SACH foot, these components can potentially have a positive effect on the asymmetry-related comorbidities analyzed in the Introduction and decrease social stigma. Further research is required to extend these results to other groups of patients, such as K2 and non-traumatic amputees. *Finally*, our results might suggest possible strategies to mitigate knee osteoarthritis of the sound side. Pending further research, transfemoral amputees might take advantage of prosthetic components with an improved knee-foot coordination to specifically tackle stance time asymmetry. Transtibial patients might benefit from improved socket construction that does not limit knee extension, and prosthetic feet with improved push-off, roll-over shape and range of motion to reduce the first peak at loading response.

## Abbreviations

GRF: Vertical component of the ground reaction force
IMS: Impulse symmetry index
P1S: Symmetry index of the first peak of the ground reaction force
SNS: Stance duration symmetry index
SPS: Step duration symmetry index
TF: Transfemoral amputees
TFC: Transfemoral amputees using a C-leg knee (Ottobock, D)
TFM: Transfemoral amputees using a mechanical knee
TT: Transtibial amputees
